# Sustained Toll-Like Receptor 9 Activation Promotes Systemic and Cardiac Inflammation, and Aggravates Diastolic Heart Failure in SERCA2a KO Mice

**DOI:** 10.1371/journal.pone.0139715

**Published:** 2015-10-13

**Authors:** Yangchen Dhondup, Ivar Sjaastad, Helge Scott, Øystein Sandanger, Lili Zhang, Solveig Bjærum Haugstad, Jan Magnus Aronsen, Trine Ranheim, Sigve Dhondup Holmen, Katrine Alfsnes, Muhammad Shakil Ahmed, Håvard Attramadal, Lars Gullestad, Pål Aukrust, Geir Christensen, Arne Yndestad, Leif Erik Vinge

**Affiliations:** 1 Research Institute of Internal medicine, Oslo University Hospital, Rikshospitalet, Oslo, Norway; 2 Center for Heart failure Research, University of Oslo, Oslo, Norway; 3 K.G. Jebsen Inflammation Research Center, University of Oslo, Oslo, Norway; 4 Institute for Experimental Medical Research, Oslo University Hospital, Ullevaal, Oslo, Norway; 5 Department of Pathology, Oslo University Hospital, Rikshospitalet, Oslo, Norway; 6 Institute of Clinical Medicine, University of Oslo, Oslo, Norway; 7 Bjørknes college, Oslo, Norway; 8 Centre for Imported and Tropical Diseases, Department of Infectious Diseases, Oslo University Hospital, Ulleval, Oslo, Norway; 9 Institute for Surgical Research, Oslo University Hospital, Rikshospitalet, Oslo, Norway; 10 Department of Cardiology, Oslo University Hospital, Rikshospitalet, Oslo, Norway; 11 Section of Clinical Immunology and Infectious Diseases, Oslo University Hospital, Rikshospitalet, Oslo, Norway; 12 Department of Internal Medicine, Diakonhjemmet Hospital, Oslo, Norway; Emory University, UNITED STATES

## Abstract

**Aim:**

Cardiac inflammation is important in the pathogenesis of heart failure. However, the consequence of systemic inflammation on concomitant established heart failure, and in particular diastolic heart failure, is less explored. Here we investigated the impact of systemic inflammation, caused by sustained Toll-like receptor 9 activation, on established diastolic heart failure.

**Methods and Results:**

Diastolic heart failure was established in 8–10 week old cardiomyocyte specific, inducible SERCA2a knock out (i.e., SERCA2a KO) *C57Bl/6J* mice. Four weeks after conditional KO, mice were randomized to receive Toll-like receptor 9 agonist (CpG B; 2μg/g body weight) or PBS every third day. After additional four weeks, echocardiography, phase contrast magnetic resonance imaging, histology, flow cytometry, and cardiac RNA analyses were performed. A subgroup was followed, registering morbidity and death. Non-heart failure control groups treated with CpG B or PBS served as controls. Our main findings were: (i) Toll-like receptor 9 activation (CpG B) reduced life expectancy in SERCA2a KO mice compared to PBS treated SERCA2a KO mice. (ii) Diastolic function was lower in SERCA2a KO mice with Toll-like receptor 9 activation. (iii) Toll-like receptor 9 stimulated SERCA2a KO mice also had increased cardiac and systemic inflammation.

**Conclusion:**

Sustained activation of Toll-like receptor 9 causes cardiac and systemic inflammation, and deterioration of SERCA2a depletion-mediated diastolic heart failure.

## Introduction

Heart failure (HF) is a major cause of morbidity and mortality worldwide with an overall prevalence of about 2–3% [1]. Although major improvements have been made in the management of HF, there is still an imminent need for novel treatment strategies. To achieve this, more knowledge of the pathogenic mechanisms is needed including identification of central pathogenic molecular players involved in the development and progression of this disorder.

Activation of the innate immune system is an important pathogenic mechanism in HF [2,3,4]. The innate immune system consists of pattern recognition receptors (PRRs) that are activated by evolutionary conserved microbial structures denominated pathogen associated molecular patterns (PAMPs). Importantly, PRRs also recognize self-antigens i.e., damage associated molecular patterns (DAMPs), which are released upon cellular stress or death [5]. Upon PRR activation, a robust inflammatory response is seen with the induction of a plethora of cytokines and adhesion molecules and subsequent mobilization of leukocytes into inflamed tissue [6].

PRRs are divided into two large families; cytosolic and membrane-bound, where Toll-like receptors (TLRs) represent the largest subfamily within the latter [7]. One particularly interesting PRR within the TLR group is Toll-like receptor 9 (TLR9). TLR9 was first identified as a PRR recognizing Cytosine-phosphate-Guanine (CpG) repeats within microbial DNA [8]. Importantly, recent data has demonstrated that endogenous mitochondrial DNA (mtDNA) is a DAMP, activating TLR9 [9,10,11]. Synthetic oligodeoxynucleotides bearing CpG motifs (CpG ODNs) activate TLR9 like unmethylated CpG motifs in bacterial DNA [12]. CpG ODNs can be classified into four classes, including type B CpG ODNs (CpG B), which are potent activators of B-cells, dendritic cells and macrophages [13]. We have recently confirmed TLR9 specificity in murine cardiac fibroblasts exposed to CpG B with or without the presence of the TLR9 inhibitor, ODN 2088 or the inhibitor of endosomal acidification, chloroquine diphosphate [14]. Moreover, others and we have demonstrated that both acute and sustained TLR9 activation with CpG B mediate systemic inflammation [15,16], and TLR9 activation has also been linked to development of myocardial failure [2,3,17]. However, the effect of sustained systemic TLR9 activation on the myocardial structure and function is not clear.

The sarco/endoplasmic reticulum Ca^2+^ ATPase (SERCA) is the nodal protein governing active diastolic function [18]. We have previously published data showing that cardiomyocyte specific deletion of SERCA2a leads to diastolic HF [19]. Although clinical diastolic HF is a multifactorial disease including abnormalities both in active relaxation and passive “stiffness” [20], abnormalities in SERCA2a function is a central entity. Thus, a murine model with cardiomyocyte specific deletion of SERCA2a can be considered a reliable model of diastolic HF *in vivo*. Experimental models have shown that altered TLR9 signalling may influence the clinical progression of *systolic* HF, though the results are ambiguous, partly reflecting differences in experimental models [2,3,17]. Thus, while systemic administration of a TLR9 agonist inhibited cardiac hypertrophy and dysfunction in isoproterenol and pressure overload-induced cardiomyopathy [3,17], TLR9 activation restricted to the cardiomyocyte leads to aggravated HF, this noteworthy also in pressure overload induced cardiomyopathy [2]. Obviously, the consequence of systemic TLR9 activation in HF is not studied at all in diastolic HF, representing a specific pathogenic entity as compared with systolic HF.

The main aim of this study was therefore to investigate the impact of sustained TLR9 stimulation using a specific TLR9 agonist, namely the CpG B, ODN 1688 in a model of murine diastolic HF i.e. SERCA2a knock out (KO).

## Methods

### 2.1. Ethics

All animals were cared for according to the Norwegian Animal Welfare Act, which conforms to the National Institutes of Health guidelines (NIH publication no. 85–23, revised 1996). Experiments were approved by the Norwegian National Animal Research Committee (FOTS application 5319). Up to six mice were kept in each cage and housed in a temperature-regulated room with a 12:12-hours day-night cycling and had free access to food and water *ad libitum*. To reduce animal suffering and distress mice were observed daily, registering morbidity and spontaneous death according to pre-specified criteria (leading to euthanization). See S1 Table. During echocardiography and PC- MRI mice were placed in a supine position on a heated pad to ensure stable conditions, anesthetized in a mixture of oxygen and 1.5–1.75% isoflurane and euthanized during deep anesthesia in a mixture of oxygen and 4–5% isoflurane. See Supporting Information for details. An experienced operator did euthanization with a quick cervical dislocation, while the remaining animals were found spontaneously dead. Five control CpG B mice lived throughout the study.

### 2.2. Induction of experimental HF and systemic inflammation

We have previously described the generation of gene-targeted mice with *C57Bl/6J* background, allowing temporal control (by tamoxifen induced expression of Cre) of cardiac myocyte specific SERCA2a gene-deletion (αMHC-MerCreMer-SERCA2a^flox/flox^) [21]. Gene-targeted mice and control mice (αMHC-MerCreMer) were generated from the same founder animals. Male mice (aged 8–10 weeks) were intraperitoneally (i.p.) injected with one single-dose of 100 μl tamoxifen [22] (T5648; Sigma Aldrich, Oslo, Norway dissolved in peanut oil to a concentration of 10 mg/ml) inducing nuclear translocation of MerCreMer, but only causing SERCA2a gene-deletion in αMHC-MerCreMer-SERCA2a^flox/flox^ mice. Four weeks after injection of tamoxifen, αMHC-MerCreMer-SERCA2a^flox/flox^ and αMHC-MerCreMer mice (hereafter denominated SERCA2a KO and controls) were randomized to receive 100μl i.p. injections of the TLR9 agonist CpG B (ODN 1668 Class B, 2μg/g body weight, Invivogen, San Diego, CA) or vehicle (PBS) every third day. Mice were divided into two substudies: 1) One cohort in which mice were followed for additional 4 weeks while receiving injections of TLR9 agonist or vehicle as described. When reaching 4 weeks after initiation of CpG B or vehicle treatment (i.e., 8 weeks after tamoxifen injection), echocardiography and phase contrast magnetic resonance imaging (PC-MRI) analyses were performed with subsequent euthanization by extirpation of the heart. Tissues (heart, lung, spleen, liver) and blood were harvested for further analyses. This renders a total duration of 8 weeks from SERCA2a gene-deletion to harvesting of organs. 2) A second cohort of mice was observed daily by an investigator blinded to genotype and intervention, registering morbidity and spontaneous death according to pre-specified criteria (leading to euthanization). Further details are provided in S1 Table.

### 2.3. Cardiac imaging

At 8 weeks after SERCA2a gene-deletion, *in vivo* heart function was evaluated by echocardiography (n = 6–12 per group) with mice placed in a supine position on a heated pad to ensure stable conditions. To keep the variations in cardiodepressive effects to a minimum, anesthesia was standardized and maintained during the procedure with a mixture of 1.75% isoflurane and 98.25% oxygen on a mask while spontaneously breathing. Echocardiographic examinations were performed with a Vevo2100 (VisualSonics, Toronto, Canada) using a 35 MHz linear array transducer (VisualSonics). Recorded data were analyzed off-line using the VEVO 2100 1.1.0 software (VisualSonics) [23]. Apart from left ventricle ejection fraction (LVEF) and left ventricle fractional shortening (LVFS) echocardiographic parameters were corrected for tibia length (TL). The duration of the procedure was no longer than 10 minutes per animal and all mice recovered from anesthesia within 1–2 min. As a supplement to echocardiographic measurements of cardiac dimensions and function, and to assess diastolic function, we performed PC-MRI using a 9.4T pre-clinical MR system (Agilent Technologies, Inc., Palo Alto, CA) with high-performance gradient (60 mm ID, rise time 130 μs, max strength 100 gauss/cm) and a quadrature volume RF coils (35 mm ID, Rapid Biomedical) dedicated to mouse imaging (n = 5–7 per group) [24]. Our group has previously assessed diastolic function in SERCA2a KO mice by the time constant of isovolumetric pressure decay (tau) [18]. However, as PC-MRI is a non-invasive method and thus more accurate and beneficial to evaluate physiological changes, we preferred to use this method for measurement of cardiac function *in vivo*. All cardiac imaging was recorded by an investigator blinded to the treatment groups. See Supporting Information for details.

### 2.4. Morphological assessment of tissue inflammation

Eight weeks after SERCA2a deletion and PC-MRI measurements, mice were euthanized during deep anesthesia in a mixture of 4–5% isoflurane and oxygen while organs (heart, lung and liver) were extirpated and rinsed in saline. A standardized 2 mm slice was taken from the hearts (n = 5–8) using a mouse heart slicer matrix (Zivic instruments, Pittsburgh, PA). The heart slice, the right lung middle lobe from hilum (n = 7–11) and the liver left lateral lobe (n = 7–10) were fixated in 4% formalin, embedded in paraffin, sectioned at 3 μm, mounted on glass slides and stained with haematoxylin and eosin (HE). A trained pathologist, blinded to the mouse genotype and intervention, visually assessed the degree of inflammation and tissue injury according to a pre-specified scoring system in hearts as well as in peripheral organs (lungs and livers). See S2 Table for details.

### 2.5. Quantification of cardiac monocyte/macrophage infiltration and fibrosis

Sections (n = 5–8 per group) of formalin-fixed and paraffin-embedded heart slices were deparaffinized by immersing slides in fresh xylene with subsequent hydration processing followed by 96°C unmasking in citrate buffer (pH 6) for 20 min. Blocking was performed using Rodent block M (Biocare Medical, Concord, CA) followed by one-hour incubation with primary antibody against macrophages (MAC-2; monoclonal rat anti-mouse, clone M3/38, IgG2A dilution 1:200; Cedarlane, Burlington, ON, Canada) at room temperature. After washing, slides were incubated 30 min with peroxidase-conjugated secondary antibody (ImmPRESS Anti-Rat Ig; Vector Laboratories, Burlingame, CA) at room temperature. Sections were developed for 8 min with chromogen for immune peroxidase staining (DAB,Vector Laboratories), before counterstaining with Haematoxylin QS (Vector Laboratories).

To quantify the amount of MAC-2 stained cells, histological slides were examined using a Nikon Eclipse E400 microscope with 40x objective. The images were automatically stitched using Hugin Panorama Photo Stitcher (Hugin 2013,http://hugin.sourceforge.net) to form a complete rendering of the slide. Prior to the analysis, the investigator was blinded to the groups. Using ImageJ (version 1.49, National Institutes of Health, Bethesda, MD) the images were thresholded using three-color channels adapted to the target stain. Manual removal of artifacts, any obvious areas of non-cardiac tissues along with the exclusion of the area of the section corresponding to the right ventricle was performed prior to measurement of the stained area. The stained area was adjusted for the total area of the section resulting in a relative quantification of the amount of MAC-2 stained cells.

As a measure of total myocardial collagen content, quantitative analysis of tissue contents of hydroxyproline was performed by HPLC using the AccQ-Fluor reagent kit (Waters Corporation Milford, MA, USA) as previously described [25].

### 2.6. RNA isolation cDNA synthesis and quantitative RT-PCR (qPCR)

Total RNA (n = 7–12 per group) was isolated from LV myocardial tissue by pre-processing with TRIzol reagent (Applied Biosystems, Foster City, CA). To ensure optimal RNA quality subsequent standard isolation using RNeasy Mini Kit (Qiagen, Venlo, The Netherlands) were performed. All RNA samples were stored at -80°C until analyzed. cDNA was synthesized using the High Capacity cDNA Reverse Transcription Kit from Applied Biosystems. PCR reactions were set up in 384 well plates using Eppendorf epMotion (Eppendorf, Hauppauge, NY). Target genes were amplified using the Power SYBR Green Master Mix (Invitrogen Life Technologies Corporation, Carlsbad, CA) and by using Applied Biosystems 7900HT Fast Real-Time PCR system. Target gene expression was normalized to glyceraldehyde 3-phosphate dehydrogenase (GAPDH). Primer sequences used for analyzing inflammatory cytokines and chemokines are provided in S3 Table.

### 2.7. Assessment of circulatory inflammatory cells

Flow cytometry (n = 6–12 per group) of circulating blood cells was performed as previously described [16]. In short, upon euthanization arterial blood (approximately 700–1000 μl) was collected at 8 weeks after SERCA2a gene deletion (by a small incision of the carotid artery) into tubes containing 50μl of EDTA 0.5M. Twenty-four hours later, 100 μl whole-blood was blocked using Mouse BD Fc Block (BD Biosciences, San Jose, CA) before labeling with 2.5μl (0.2 mg/ml) CD11b-APC and Ly6G-PE or 1.0μl (0.5mg/ml) CD3e-FITC with subsequent lysis of red blood cells. Flow cytometry analysis was performed blinded to the treatment groups, using FACSCalibur (BD Biosciences).

### 2.8. Statistical analyses

Unpaired data were evaluated using Graphpad Prism 6 (GraphPad, San Diego, CA), ANOVA Kruskal Wallis test, and subsequent Mann-Whitney non-parametric test for comparison of two groups. Survival analysis was performed using Log rank (Mantel Cox test). Results are shown as mean±SEM. To compare the distribution of score numbers between the groups after scoring inflammation in heart-, lung- and liver tissue, we used IBM SPSS Statistics (version 22) Chi square test. Probability values of *P*<0.05 were considered significant.

## Results

### 3.1. Systemic TLR9 activation leads to premature death in SERCA2a KO induced diastolic HF

**Fig 1:**
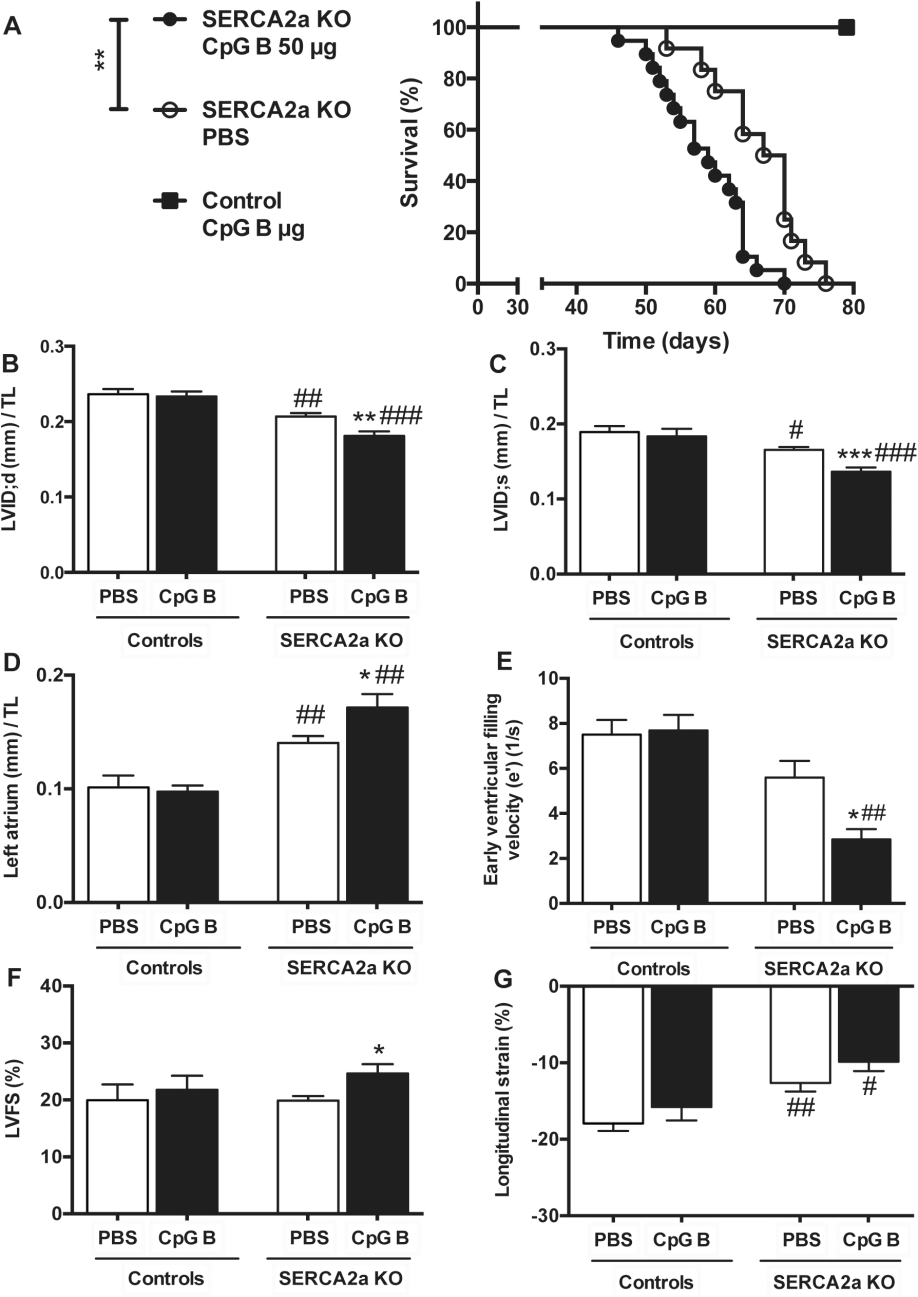
Increased mortality and deteriorated cardiac functions and structures in TLR9 stimulated (CpG B) SERCA2A KO mice 8 weeks after gene excision and 4 weeks after initiation of sustained TLR9 stimulation. (A) Survival analysis: median 59 days in TLR9 stimulated SERCA2a KO mice vs. 64.5 days in SERCA2a KO. Groups were compared using Log rank (Mantel Cox test, n = 16–19 per group, n = 5 in TLR9 stimulated control group). (B) LV inner diastolic diameter/TL (LVID;d, mm). (C) LV inner systolic diameter/TL (LVID;s, mm). (D) Left atrium/TL (mm). (E) Early ventricular filling velocity (e’) (F) LV fractional shortening (LVFS%) (G) Longitudinal strain (%). LVID (B and C), left atrium (D) and LVFS (F) were determined using echocardiography (n = 6–12 per group), and e’ (E) and longitudinal strain (G) were determined using PC-MRI (n = 5–7 per group). TL, tibia length (mm). Statistics were done using Mann Whitney U- test. Data are mean± SEM. *P<0.05, **P <0.01, ***P<0.001 vs. SERCA2a KO mice. ^#^ *P*<0.05, ^##^ *P*<0.01, ^###^ *P*<0.001 vs. control with same intervention.

All the animals reached our pre-specified end-parameter (death or euthanasia according to pre-specified criteria) after induction of cardiomyocyte-specific deletion of the SERCA2a gene. Of the 40 mice (SERCA2a KO PBS n = 16; SERCA2a CpG B n = 19; Control CpG B n = 5), 9 animals were euthanized due to objective pre-specified criteria of distress indicating severe HF (SERCA2a KO PBS n = 2; SERCA2a KO CpG B n = 7). There was a non-significant trend towards a higher proportion of CpG B treated SERCA2a KO mice euthanized (p = 0.13, Fischer’s exact test). We found reduced life expectancy in TLR9 stimulated SERCA2a KO mice compared with PBS treated SERCA2a KO mice (median 59 vs. 64.5 days, respectively, p = 0.004). Importantly, this was also significant when excluding mice that were euthanized (p<0.01). Within the observation period, there was no mortality or clinical morbidity in TLR9 stimulated control mice (Fig 1A).

### 3.2. TLR9 stimulation deteriorated diastolic function in SERCA2a KO mice

Echocardiography and PC-MRI was performed 4 weeks after initiation of CpG B or vehicle treatment and 8 weeks after cardiomyocyte-specific deletion of the SERCA2a gene. Results are displayed in Tables 1 and 2. As the impairment of active relaxation caused by SERCA2a depletion commences, the ability to increase chamber size during diastole is severely restrained causing a lower LV inner diastolic diameter (LVID;d), subsequently necessitating a lower LV inner systolic diameter (LVID;s) in attempt to maintain stroke volume. Upon sustained TLR9 stimulation, these parameters were significantly worsened (Fig 1B and 1C). Another major echocardiographic phenotype of SERCA2a KO mice is enlargement of the left atrium (LA), a consequence of the diastolic HF causing increased LV filling pressure. In this study, TLR9 stimulated SERCA2a KO mice had significantly larger LA compared with PBS treated SERCA2a KO mice, further suggesting aggravation of the diastolic impairment (Fig 1D). In corroboration with these results, PC-MRI analyses showed decrease in the early ventricular filling velocity (e’) in SERCA2a KO mice, which was significantly worsened when challenged with sustained TLR9 stimulation (Fig 1E). In contrast to the worsening of diastolic function, TLR9 activation resulted in increased LVEF (Table 1) and LVFS (Fig 1F). The latter are observations we have described previously and suggested partly being mediated by altered intracellular contents of sodium [19]. Indeed, in other cardiac clinical conditions associated with reduced cardiac dimensions (like hypertrophic cardiomyopathy or severe aortic stenosis), increased LVFS and LVEF are seen [19,21]. In fact, long axis strain, assessed by PC-MRI, showed that SERCA2a KO mice in the later phases of HF development (8 weeks) display reduced axial shortening (Fig 1G), albeit with no significant effect of TLR9 activation. There were no significant differences in heart and body weights between the groups (S4 Table). However, CpG B treated SERCA2a KO mice demonstrated increased relative wall thickness (RWT) (Table 1). As the SERCA 2a KO phenotype is driven by an active relaxation deficit, the RWT does not reflect actual hypertrophy but in fact a reduction in LVID;d due to a reduction of the heart size in total. Thus, the impairments in cardiac function promoted by sustained TLR9 stimulation in SERCA2a KO mice seem to be predominantly driven by aggravations in diastolic function.

**Table 1:** Echocardiographic parameters in SERCA2a KO and control mice 8 weeks after gene excision and 4 weeks after initiation of sustained TLR9 stimulation.

	Controls		SERCA2a KO	
	PBS (n = 6)	CpG B (n = 8)	PBS (n = 12)	CpG B (n = 9)
LVEF (%)	40.5 ± 12.4	43.7 ± 12.0	41.2 ± 5.0	49.6 ± 8.0 *
CO (ml/min)/ TL	1.4 ± 0.2	2.2 ± 0.8	1.0 ± 0.3	1.0 ± 0.6 ^##^
SV (ml)/TL	3.2 ± 0.1	4.0 ± 1.1	2.3 ± 0.6 ^###^	1.9 ± 0.7 ^##^
IVS;d (mm)/TL	0.05 ± 0.005	0.04 ± 0.009	0.04 ± 0.004	0.05± 0.006
IVS;s (mm)/TL	0.06 ± 0.009	0.06 ± 0.010	0.05 ± 0.007	0.06 ± 0.007
LVPW;d (mm)/TL	0.05 ± 0.010	0.04 ± 0.006	0.05 ± 0.010	0.04 ± 0.006
LVPW;s (mm)/TL	0.06 ± 0.014	0.05 ± 0.008	0.054 ± 0.008	0.06 ± 0.010
LVvol;d (μL)/TL	4.9 ± 0.8	4.6 ± 0.6	3.6 ± 0.5	2.7 ± 0.6 *
LVvol;s (μL)/TL	3.0 ± 0.8	2.6 ± 0.8	2.1 ± 0.3	1.4 ± 0.4 *
RWT	0.39 ± 0.038	0.37 ± 0.042	0.43± 0.064	0.51 ± 0.105 ^##^
Heart rate (BPM)	467.4 ± 55.8	488.6 ± 54.5	452.5 ± 44.2	428.4 ± 57.6 ^#^

LV, Left ventricle; EF, Ejection fraction; CO, Cardiac output; SV, Stroke volume; d, diastolic; s, systolic; TL, Tibia length (mm); IVS, inter ventricular septum thickness; LVPW, LV posterior wall thickness; LVvol, LV volume; RWT, relative wall thickness (Formula: IVS;d+LVPW;d/ LVID;d); Data are expressed as the mean±SD.

*P<0.05 vs. SERCA2a KO mice.

^#^ *P*<0.05

^##^ *P*<0.01

^*###*^ *P*<0.001 vs. control with same intervention.

**Table 2:** Phase contrast magnetic resonance imaging in SERCA2A KO and control mice 8 weeks after gene excision and 4 weeks after initiation of sustained TLR9 stimulation.

	Controls		SERCA2a KO	
	PBS (n = 6–8)	CpG B (n = 7–8)	PBS (n = 7–8)	CpG B (n = 5–6)
Late ventricular filling velocity;a’ (1/s)	5.9 ± 2.4	3.8 ± 1.0	4.8 ± 2.0	3.5 ± 0.5
Long.strain rate;s (1/s)	-4.9 ± 0.8	-4.7 ± 1.3	-4.1 ± 1.1	-3.7 ± 1.1
Max.rad velocity (cm/s)	1.0 ± 0.5	1.3 ± 0.3	1.0 ± 0.3	0.81 ± 0.16
Min.rad velocity (cm/s)	-1.6 ± 0.3	-1.7 ± 0.4	0–1.2 ± 0.2	-1.0 ± 0.3
Circ.strain (%)	-14.9 ± 2.3	15.2 ± 2.9	-12.0 ± 1.6	-9.3 ± 3.4

Long.strain rate, Longitudinal strain rate; Max.rad velocity, Maximum radial velocity; Min. Rad velocity, Minimum radial velocity; Circ.strain, Circumferential strain. Data are expressed as mean±SD.

### 3.3. Sustained TLR9 stimulation augments cardiac inflammation and fibrosis in SERCA2a KO induced HF

**Fig 2:**
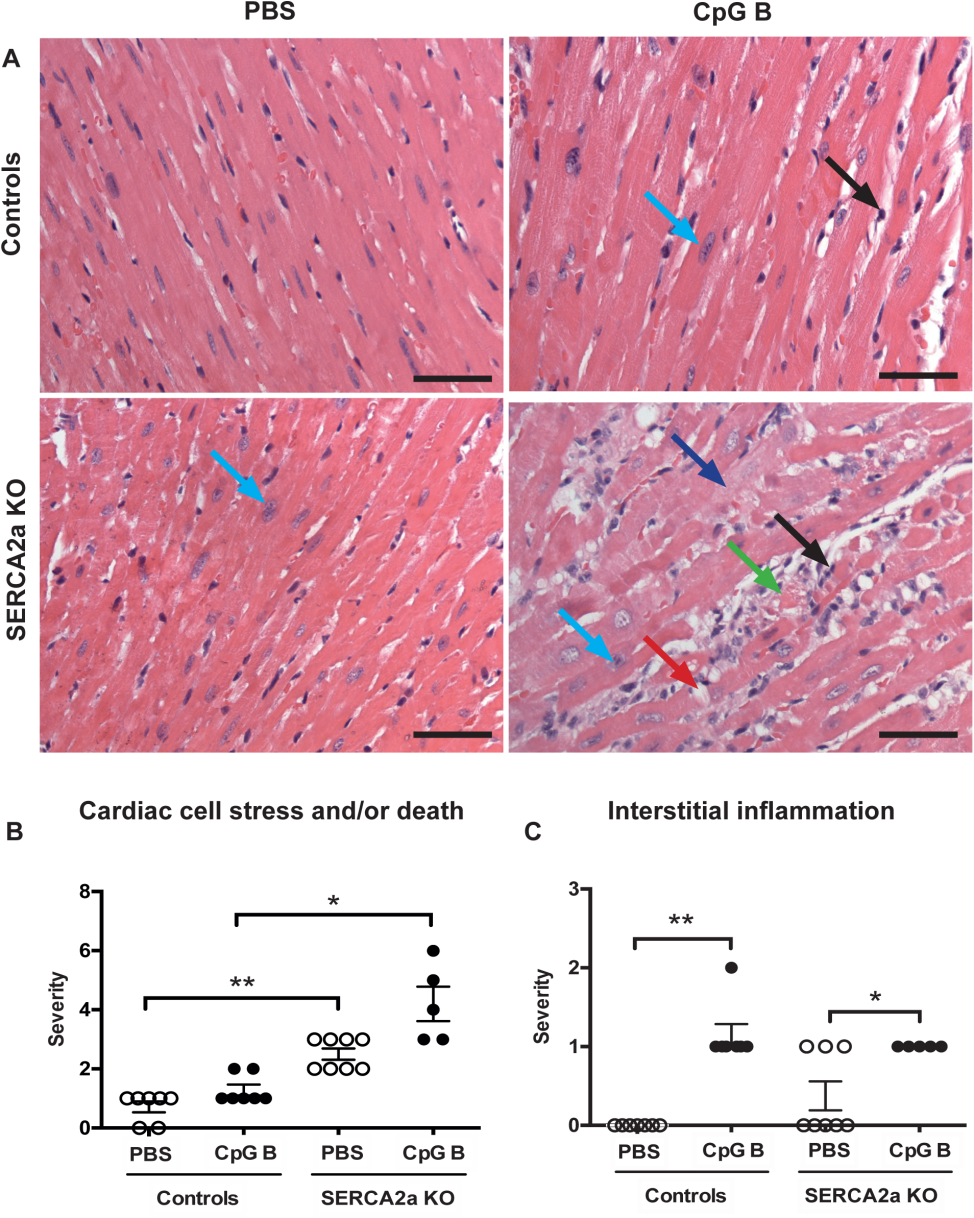
Histology of haematoxylin and eosin stained hearts. (A) Photos taken with 40x objective (scale bar 50μm). (A-B) *Cardiac cell stress and/or death*: increased nucleus-to-cytoplasm ratio (light blue arrow), swollen cardiomyocyte cytoplasm (dark blue arrow), vacuolization (red arrow), cardiac cell death (green arrow). (A-C) *Interstitial leukocyte infiltration* (black arrow). See S2 Table for details. Distribution between the groups was compared using Chi-square test (n = 5–8 per group). *P<0.05, **P<0.01 vs. SERCA2a KO mice.

**Fig 3:**
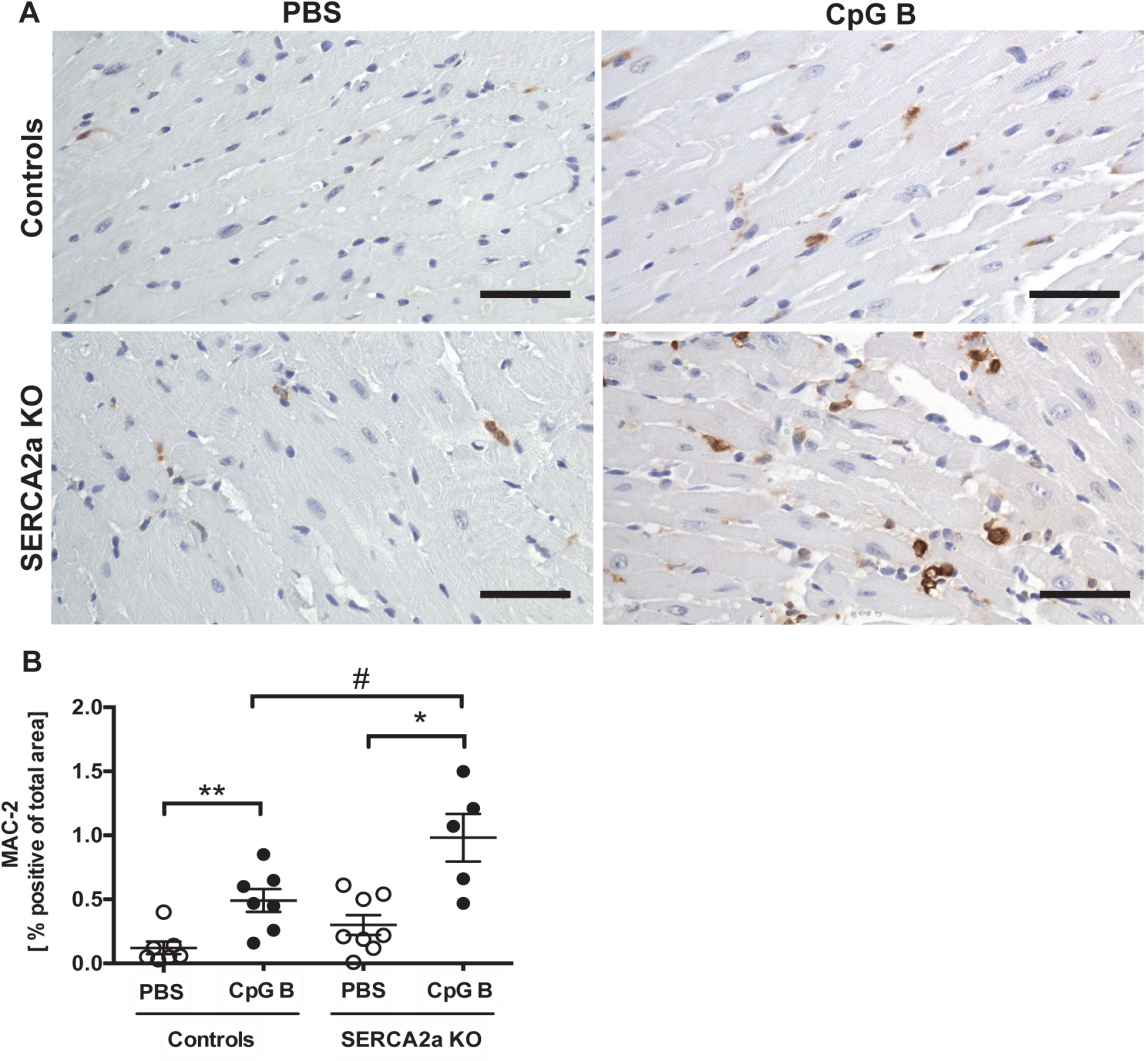
Increased number of MAC-2 positive cells in TLR9 stimulated mouse hearts. (A) Photos taken with 40x objective (scale bar 50μm). (B) MAC-2 image based quantification of MAC-2 positive cells. Statistics were done using Mann Whitney U- test (n = 5–8 per group). Lines and error bars are mean±SEM. *P<0.05, **P<0.01 vs. SERCA2a KO mice. ^#^ *P*<0.05 vs. control with same intervention.

**Fig 4:**
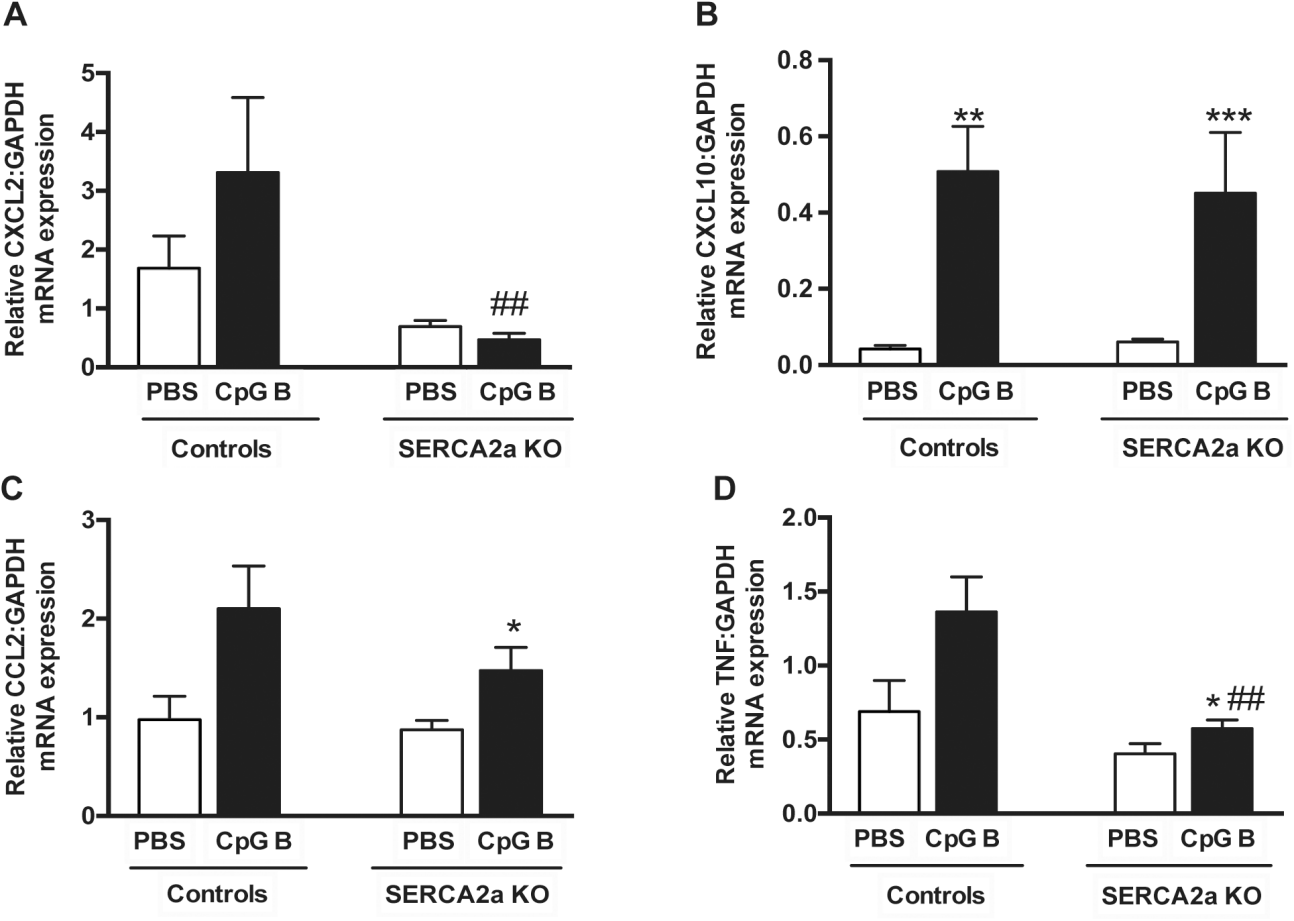
Quantitative PCR on left ventricle myocardial tissue of mice with HF 8 weeks after SERCA2a gene excision and 4 weeks after initiation of sustained TLR9 stimulation. (A) CXCL2, Chemokine C-X-C motif ligand 2 (B) CXCL10, Chemokine C-X-C motif ligand 10 (C) MCP-1, Monocyte chemotactic protein-1 (D) TNF, Tumor necrosis factor. Statistics were done using Mann Whitney U- test (n = 7–12 per group). Data are mean±SEM. *P<0.05, **P<0.01 vs. SERCA2a KO. ^#^ *P*<0.05, ^##^ *P*<0.05 vs. control with same intervention.

Cardiac inflammation was assessed by histopathological staging, quantification of cardiac monocyte/macrophage infiltration and by assessing cytokine gene expression. The histopathological staging was performed using a simple three/four-point score visual assessment of cardiac myocyte stress and or/death (nuclear-to-cytoplasm ratio and vacuolization or necrotic muscle fibers) as well as quantification of cardiac leukocyte infiltration. Several significant findings were revealed. First, signs of cardiac myocyte stress and/or death were seen in TLR9 stimulated control mice. Also, a higher degree of this phenomenon could be seen in SERCA2a KO hearts compared to PBS control mice. Although, the combination of SERCA2a KO and TLR9 stimulation appeared additive, it was not significantly different to SERCA2a KO alone (Fig 2A and 2B). Second, TLR9 stimulation increased leukocyte infiltration as assessed by primarily lymphocytes in both control and SERCA2a KO mice, with no difference between the genotypes (Fig 2C). Third, in contrast to lymphocyte infiltration, a significantly higher cardiac infiltration of monocytes/macrophages was seen in TLR9 stimulated SERCA2a KO mice compared with both TLR9 stimulated control mice and PBS treated SERCA2a KO mice (Fig 3A and 3B). Finally, whereas SERCA2a KO hearts did not display significant alterations in the chosen inflammatory genes, sustained TLR9 stimulation induced a significant up-regulation of CXCL10, CXCL2, CCL2 and TNF in these mice (Fig 4A–4D).

**Fig 5:**
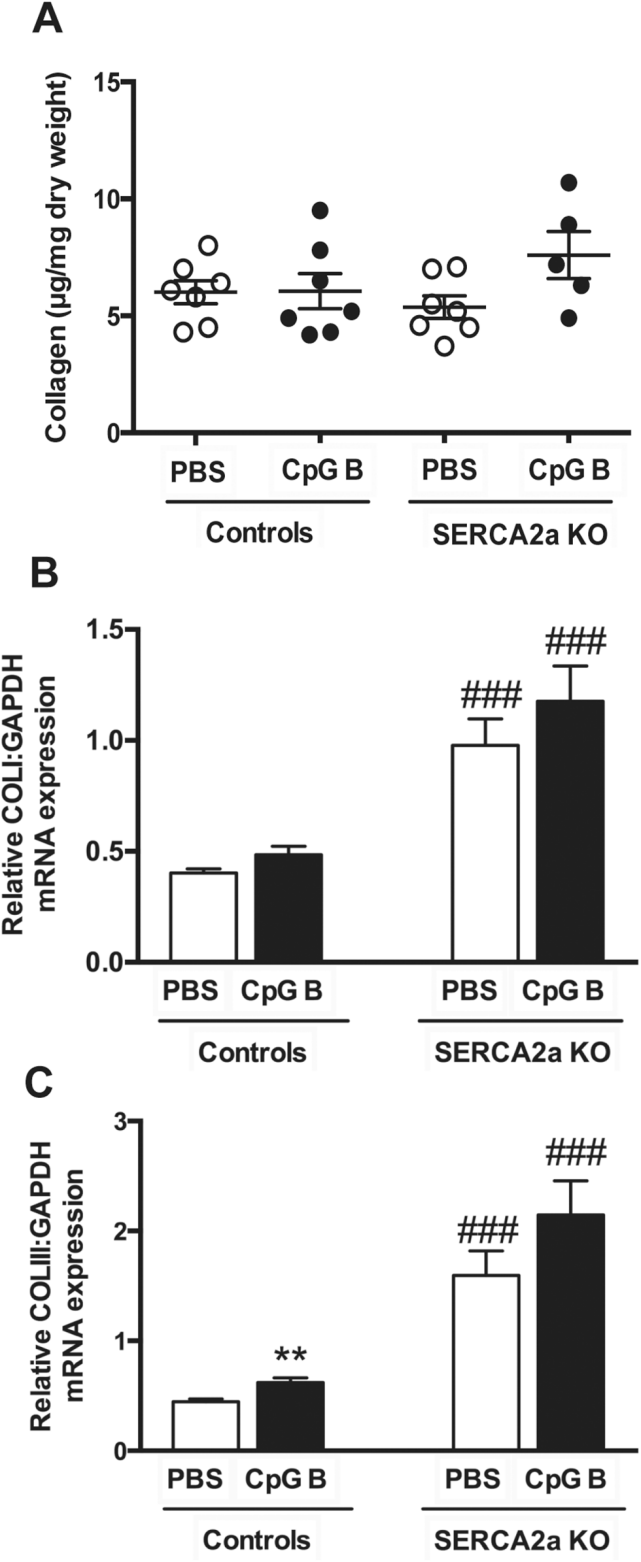
Hydroxyproline analysis by HPLC and mRNA gene expressions of Collagen I and III demonstrated a trend towards increased collagen deposition in TLR9 stimulated SERCA2a KO hearts. (A) Collagen (μg/ml dry weight) (B) Collagen I (C) Collagen III. Statistics were done using Mann Whitney U- test (n = 7 per group except n = 5 in CpG SERCA2a KO group). Lines and error bars are mean±SEM. **P<0.01 vs. SERCA2a KO mice. ^###^ *P*<0.0001 vs. controls with same intervention.

As increased inflammatory cells were detected in TLR9 stimulated hearts, we assessed cardiac fibrosis as a potential cause of diastolic dysfunction. Measurements of hydroxyproline in LV myocardial tissue samples of TLR9 stimulated SERCA2a KO hearts demonstrated a trend towards increased collagen deposition (p = 0.07). This trend, however, was supported by increased collagen I (p = 0.19) and III (p = 0.07) mRNA gene expressions (Fig 5).)

Taken together, these data do provide evidence of cardiac inflammation upon sustained, systemic TLR9 stimulation with enhanced monocyte/macrophage infiltration and possibly increased fibrosis in TLR9 stimulated SERCA2a KO mice as a pathomechanistic explanation for the deteriorated diastolic function.

### 3.4. Both HF and sustained TLR9 stimulation promote systemic inflammation

**Fig 6:**
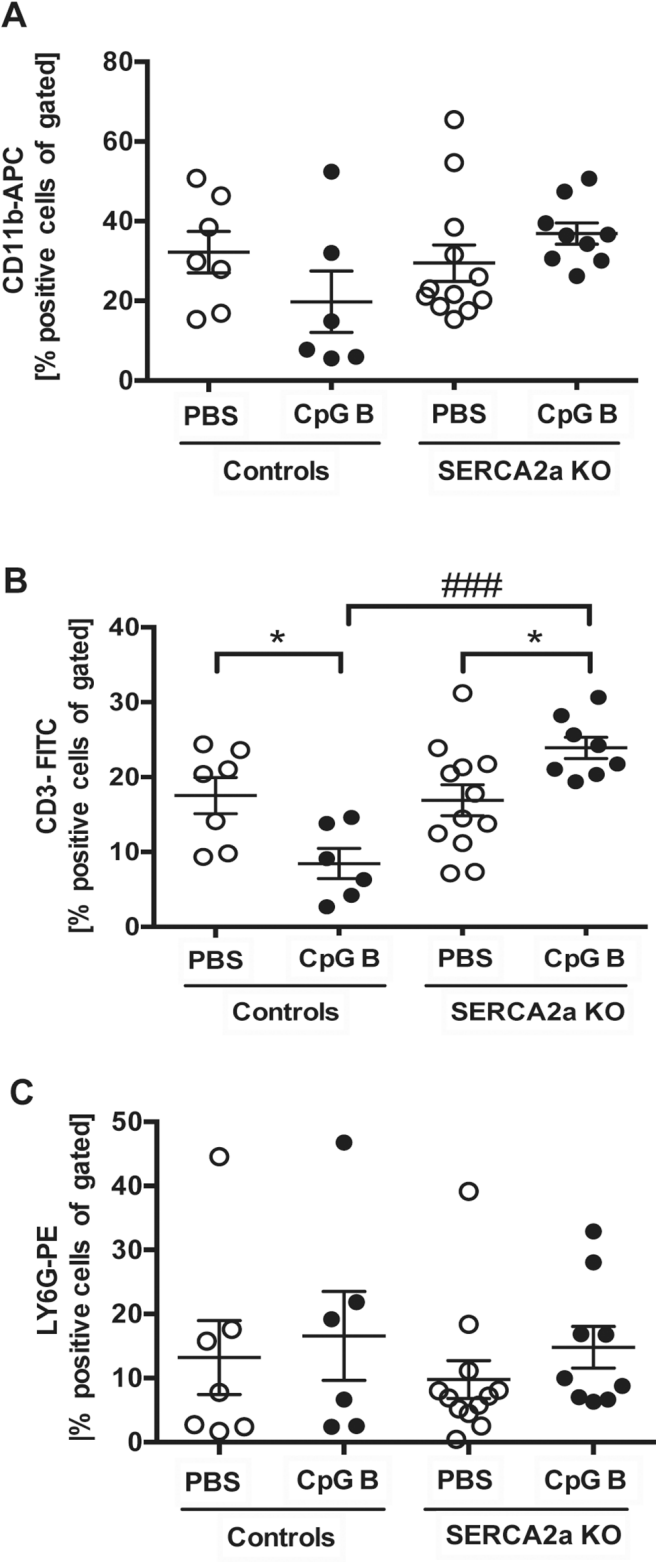
Flow cytometry of CD11b, CD3 and LY6G stained circulating blood cells showed alterations in distribution of cells. (A) CD11b-APC positive cells (B) CD3-FITC positive cells and (C) LY6G-PE positive cells. Statistics were done using Mann Whitney U- test (n = 6–12 per group). Lines and error bars are mean±SEM. *P<0.05 vs. SERCA2a KO mice. ^###^ *P*<0.001 vs. control with same intervention.

Both HF (SERCA2a KO) [2] and repetitive administrations of a TLR9 agonist (i.e., CpG B) could promote inflammation in non-cardiac organs [15]. Thus, we assessed the inflammatory responses in lung, liver and circulatory immune cells in mice with combinations of those two conditions. Using flow cytometry, we analyzed circulating levels of monocytes (CD11b positive cells), T cells (CD3 positive cells) and granulocytes (Ly6G positive cells) and found no significant alterations in SERCA2a KO mice compared to controls (Fig 6A–6C). As previously reported [15], sustained TLR9 stimulation induced leukopenia with lower amounts of CD11b and CD3 positive cells in TLR9 stimulated control mice (Fig 6A and 6B). In contrast, TLR9 stimulated SERCA2a KO mice showed a higher level of CD11b (p = 0.06) and CD3 (p = 0.03) positive cells than seen in both PBS treated SERCA2a mice and TLR9 stimulated control mice (Fig 6A and 6B). As for the distribution of Ly6G positive cells, no significant differences could be seen between any groups (Fig 6C).

**Fig 7:**
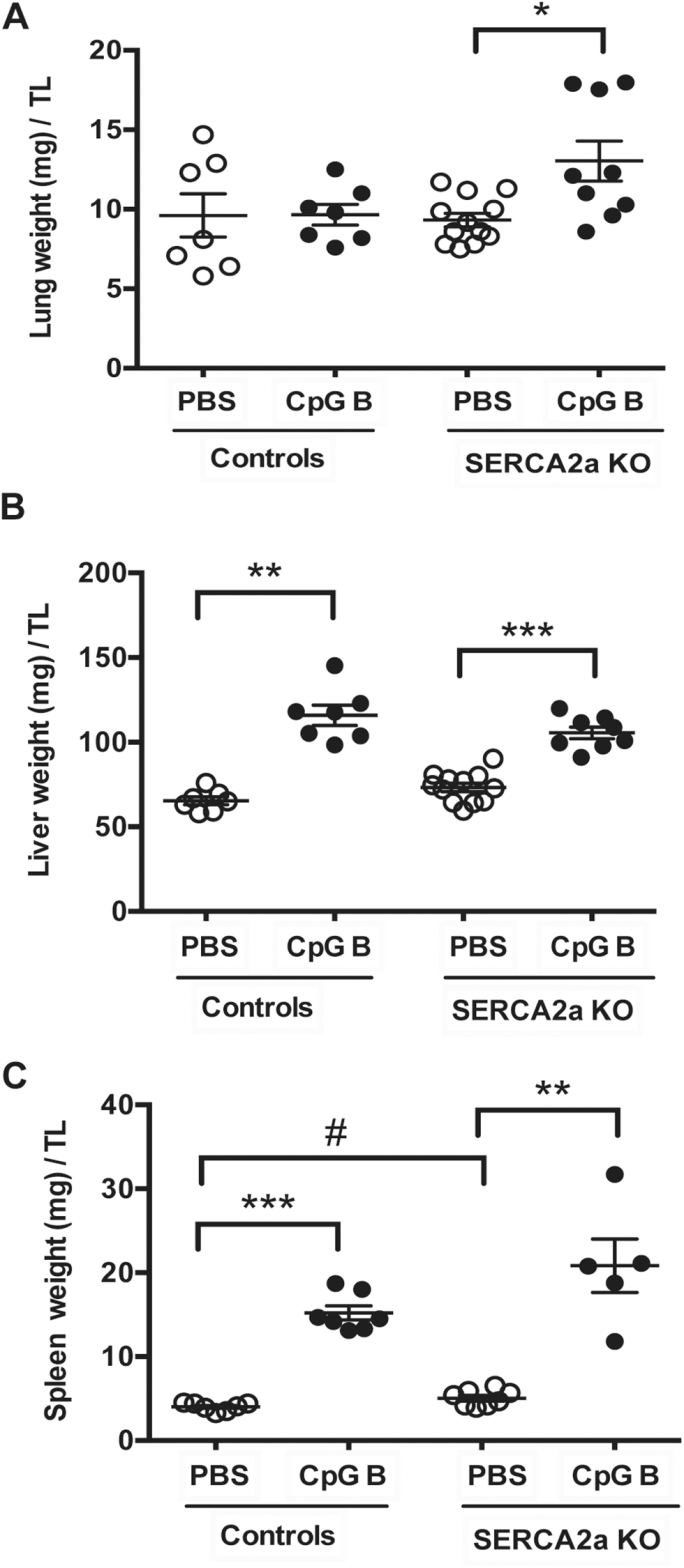
Organ weights of mice at 8 weeks after SERCA2A gene excision and 4 weeks after initiation of sustained TLR9 stimulation. (A) Lung weight (mg)/TL (B) Liver weight (mg)/TL (C) Spleen weight (mg)/TL. TL, tibia length (mm). Statistics were done Mann Whitney U- test (n = 7–12 per group). Data are meanxSEM. *P<0.05, **P<0.01, **P <0.001 vs. SERCA2a KO. ^#^ *P*<0.05 vs. control with same intervention.

Histopathological staging, using pre-defined histological criteria as viewed in S2 Table, was used to assess degree of liver and lung inflammation. Even though we did find a significantly higher wet lung weight in SERCA2a KO mice challenged with sustained TLR9 stimulation, no effect of CpG B stimulation was evident in histopathological staining in either SERCA2a KO or control mice. We did not see any histopathological signs of lung edema. A robust pulmonary inflammatory response, however, could be seen in SERCA2a KO mice with no further effect of TLR9 (S1 Fig, Fig 7A). In contrast, histopathological staging of liver inflammation revealed a significant alteration in mice challenged with TLR9 stimulation with increased lymphocytes in both SERCA2a KO and control mice (S2 Fig, S3 Fig), accompanied by significantly increased liver weights (Fig 7B).

## Discussion

Most studies on HF focus on aspects related to intrinsic cardiac mechanisms of evolving heart dysfunction. However, HF patients often suffer from co-morbidities with inflammation as the predominant feature. Hypothetically, triggers of such systemic inflammatory states and/or the consequence of systemic inflammation may impact the natural progression of HF. In this study we show that persistent systemic TLR9 stimulation induces increased mortality and worsening of diastolic function in a model of diastolic HF (i.e., cardiomyocyte specific SERCA2a KO). These features were accompanied by enhanced monocyte/macrophage infiltration and expression of inflammatory cytokines, and a trend towards increased collagen deposition within the failing myocardium, as well as increased levels of monocytes and T cells in peripheral blood in TLR9 stimulated SERCA2a KO mice. Our findings suggest a link between systemic inflammation and worsening of diastolic HF, potentially involving increased monocyte/macrophage driven myocardial inflammation.

Our major finding was that SERCA2a KO mice challenged with sustained TLR9 stimulation died prematurely accompanied by worsening of diastolic dysfunction compared with PBS treated SERCA2a KO mice. We believe our data supports this being a consequence of cardiac death due to 1) no mortality in TLR9 stimulated control mice and 2) clear evidence of deteriorated cardiac dysfunction in TLR9 stimulated SERCA2a KO mice. Meticulous analyses of cardiac function by echocardiography and PC-MRI lead us to conclude that the predominant cause of TLR9 stimulated aggravation of HF is worsening of diastolic function. The SERCA2a KO mice in the late phases do display evidence of systolic impairment, but no significant worsening of this dysfunction could be seen upon sustained TLR9 stimulation. In fact, CpG B stimulated SERCA2a KO mice showed higher LVEF in an early phase. LVFS and LVEF are known to be rather insensitive parameters to smaller alterations in systolic dysfunction, whereas PC-MRI is considered a more sensitive method [26], in particular by measurement of LV longitudinal strain. Thus, the SERCA2a KO mice in addition to diastolic dysfunction, had systolic dysfunction.

The worsened diastolic HF could be a consequence of impaired active relaxation and/or passive recoil of the myocardium [15]. Our model of diastolic HF is one predominately conveyed by interference in active relaxation [17]. Accordingly, it is reasonable to suggest that the premature deaths of TLR9 stimulated mice are a consequence of an accelerated reduction in diastolic function. Our finding of a trend towards increased tissue fibrosis in CpG B treated mice, as well as increased inflammatory responses, suggests that the reduced diastolic function also involves deteriorated passive relaxation. However, the mechanisms leading to deteriorated diastolic heart failure are still elusive, as our model of chronic systemic TLR9 stimulation does not allow discrimination between cardiac TLR9 stimulation towards the indirect effects of systemic TLR9 stimulation and subsequent effects on the heart caused by the following systemic inflammatory response.

Both scenarios are possible. On the one hand, stimulation of cardiac TLR9 does impact contractile performances on the cardiac myocyte level [27,28]. No studies however, have specifically addressed the significance of TLR9 stimulation on active relaxation. Still, a recent study linked non-canonical TLR9 signaling to SERCA2 function through CpG B induced reduction of SERCA2 function and cell survival [29]. On the other hand, stimulation of both cardiac and non-cardiac TLR9 will elicit signaling leading to activation of NF-kB and Interferon regulatory factor 3/7 (IRF3/7), both resulting in release of various inflammatory cytokines and chemokines. Indeed, in the present study we found that the impairment of diastolic function was associated with increased myocardial inflammation with enhanced expression of inflammatory cytokines and increased infiltration of monocytes/macrophages. This could point to an inflammatory phenotype within the myocardium that affects its function.

While systemic administration of TLR9 agonist has been shown to attenuate myocardial hypertrophy and dysfunction in both isoproterenol and pressure overload-induced cardiomyopathy [3,17], we found worsening of diastolic function in a model of diastolic HF with no attenuating effects on systolic function. The studies of Yang and Velten, however, have designs that substantially differ from our study, as their mode of TLR9 stimulation was restricted to the time period prior to intervention, potentially preconditioning the heart to myocardial damage. Also, it is not inconceivable that systemic TLR9 activation have different effects on systolic and diastolic HF. HF with preserved systolic function is an increasing problem in clinical cardiology. About 50% of patients with HF have preserved ejection fraction (HFpEF), which is especially common in elderly people with highly prevalent co-morbid conditions [30,31]. Our findings may suggest a role for TLR9 activation in the pathogenesis of this disorder.

In the present study we found that systemic TLR9 activation affected various organ systems. In lungs, only minor effects were seen with sustained TLR9 stimulation. In contrast, the inflammatory responses seen in liver were predominately driven by TLR9 stimulation with no additional effect of diastolic HF. In the myocardium, however, there was a tendency towards increased inflammatory responses in the TLR9 stimulated SERCA2a KO mice as compared to the pure SERCA2a KO and TLR9 stimulated control mice, indicating an interaction between TLR9 and HF within the myocardium. Such an interaction was even more evident in peripheral blood. While the levels of T cells and monocytes were attenuated upon TLR9 stimulation in control mice, the proportion of these cells increased in TLR9 stimulated SERCA2a KO mice. Recent studies suggest an important role for spleen-derived monocytosis with subsequent increased cardiac macrophage infiltration during myocardial remodeling following an ischemic event [32]. It has also been proposed that a similar mechanism could be in play during HFpEF [33]. If so, TLR9-driven mechanisms could be involved, although this will have to be proved in forthcoming studies.

Even though our data demonstrates that sustained systemic TLR9 stimulation aggravates diastolic HF in our model of gene-targeted diastolic HF, there are several limitations as to mechanistic explanations of causality, as well as extrapolations to clinical inflammatory disease states and other HF conditions. First, our pharmacological inflammatory model does not allow discrimination between effects caused by direct cardiac TLR9 stimulation to that of indirect effects mediated by systemic inflammation. Second, although several systemic inflammatory conditions have disturbances in the innate immune system as important features, and some of these again specifically encompassing distorted TLR9 signaling [34], sustained TLR9 stimulation does not necessarily represent a clinically relevant inflammatory condition. Finally, the cardiac myocyte SERCA2a KO model does not adequately represent the molecular basis for, or the clinical features of, diastolic HF.

With the above-mentioned limitations, our study suggests a link between systemic TLR9 activation and diastolic HF, involving systemic inflammation and increased monocyte/macrophage infiltration within the failing myocardium. These data do provide a platform for future investigations studying both systemic and myocardial restricted TLR9 activation combined with various models of experimental HF.

## Supporting Information

S1 Fig Histology of haematoxylin and eosin stained lungs. (A) Photos taken with 40x objective (scale bar 50μm). (A-B) *Vascular inflammation*: intima inflammation (yellow arrow = thickening of intima, black arrow = leukocytes). *Alveolar inflammation* (A and C, black arrows = leukocytes). See S2 Table for details. Distribution between the groups was compared using Chi-square test (n = 7–11 per group). ^#^ *P*<0.05, ^##^ *P*<0.01 vs. control with same intervention.
(DOC)

S2 Fig Histology of haematoxylin and eosin stained liver. (A) Photos taken with 40x objective (scale bar 50μm). *Portal inflammation* (A-B, black arrows = leukocytes). *Lobular inflammation* (A and C, black arrows = leukocytes). See S2 Table for details. Distribution between the groups was compared using Chi-square test (n = 7–10 per group). *P<0.05, **P<0.01 vs. SERCA2a KO mice.
(DOC)

S3 Fig Total score of inflammation in haematoxylin and eosin stained hearts, lungs and livers. Combined data from Fig 2, S1 and S2 Figs. See S2 Table for details. (A) Total score of inflammation in hearts (n = 5–8 per group). (B) Total score of inflammation in lungs (n = 7–11 per group). (C) Total score of inflammation in livers (n = 7–10 per group). Distribution between the groups was compared using Chi-square test. *P<0.05, **P<0.01 vs. SERCA2a KO mice. ^#^ *P*<0.05, ^##^ *P*<0.05 vs. control with same intervention.
(DOC)

S1 Methods Phase contrast magnetic resonance imaging (PC-MRI). (DOC)

S1 Table Pre-specified criteria for evaluating morbidity and spontaneous death. (DOC)

S2 Table Eight weeks after gene excision and 4 weeks after initiation of sustained TLR9 stimulation, the degree of inflammation in hearts, lungs and livers were scored by a pathologist blinded to genotype and intervention. (DOC)

S3 Table Primer sequences used in RT PCR analyses. (DOC)

S4 Table Physiological parameters in SERCA2a KO and control mice 8 weeks after gene excision and 4 weeks after initiation of TLR9 stimulation. (DOC)
