# Supplementary material for: Sustained Toll-Like Receptor 9 Activation Promotes Systemic and Cardiac Inflammation, and Aggravates Diastolic Heart Failure in SERCA2a KO Mice
Source: PLoS One. 2015 Oct 13;10(10):e0139715. doi: 10.1371/journal.pone.0139715 (PMC4604200; doi:10.1371/journal.pone.0139715)
Supplement: S1 Fig — (A) Photos taken with 40x objective (scale bar 50μm). (A-B) Vascular inflammation: intima inflammation (yellow arrow = thickening of intima, black arrow = leukocytes). Alveolar inflammation (A and C, black arrows = leukocytes). See S2 Table for details. Distribution between the groups was compared using Chi-square test (n = 7–11 per group). # P<0.05, ## P<0.01 vs. control with same intervention. (DOC) [file pone.0139715.s001.doc]

# Supporting Figure Captions

**S1 Fig. Histology of haematoxylin and eosin stained lungs.**

**(**A) Photos taken with 40x objective (scale bar 50µm). (A-B) *Vascular inflammation:* intima inflammation (yellow arrow= thickening of intima, black arrow= leukocytes). *Alveolar inflammation* (A and C, black arrows= leukocytes). See S2 Table for details. Distribution between the groups was compared using Chi-square test (n= 7-11 per group). ^#^*P*<0.05, ^##^*P*<0.01 vs. control with same intervention.
