# Supplementary material for: Sustained Toll-Like Receptor 9 Activation Promotes Systemic and Cardiac Inflammation, and Aggravates Diastolic Heart Failure in SERCA2a KO Mice
Source: PLoS One. 2015 Oct 13;10(10):e0139715. doi: 10.1371/journal.pone.0139715 (PMC4604200; doi:10.1371/journal.pone.0139715)
Supplement: S2 Fig — (A) Photos taken with 40x objective (scale bar 50μm). Portal inflammation (A-B, black arrows = leukocytes). Lobular inflammation (A and C, black arrows = leukocytes). See S2 Table for details. Distribution between the groups was compared using Chi-square test (n = 7–10 per group). *P<0.05, **P<0.01 vs. SERCA2a KO mice. (DOC) [file pone.0139715.s002.doc]

# Supporting Figure Captions

**S2 Fig. Histology of haematoxylin and eosin stained liver.**

**(**A) Photos taken with 40x objective (scale bar 50µm). *Portal inflammation* (A-B, black arrows= leukocytes). *Lobular inflammation* (A and C, black arrows= leukocytes). See S2 Table for details. Distribution between the groups was compared using Chi-square test (n= 7-10 per group). **P*<0.05, ***P*<0.01 vs. SERCA2a KO mice.
