# Supplementary material for: Sustained Toll-Like Receptor 9 Activation Promotes Systemic and Cardiac Inflammation, and Aggravates Diastolic Heart Failure in SERCA2a KO Mice
Source: PLoS One. 2015 Oct 13;10(10):e0139715. doi: 10.1371/journal.pone.0139715 (PMC4604200; doi:10.1371/journal.pone.0139715)
Supplement: S3 Fig — Combined data from Fig 2, S1 and S2 Figs. See S2 Table for details. (A) Total score of inflammation in hearts (n = 5–8 per group). (B) Total score of inflammation in lungs (n = 7–11 per group). (C) Total score of inflammation in livers (n = 7–10 per group). Distribution between the groups was compared using Chi-square test. *P<0.05, **P<0.01 vs. SERCA2a KO mice. # P<0.05, ## P<0.05 vs. control with same intervention. (DOC) [file pone.0139715.s003.doc]

# Supporting Figure Captions

**S3 Fig. Total score of inflammation in haematoxylin and eosin stained hearts, lungs and livers. Combined data from Fig. 2, S1 and S2 Figs. See S2 Table for details**.

(A) Total score of inflammation in hearts (n= 5-8 per group). (B) Total score of inflammation in lungs (n= 7-11 per group). (C) Total score of inflammation in livers (n= 7-10 per group). Distribution between the groups was compared using Chi-square test. **P*<0.05, ***P*<0.01 vs. SERCA2a KO mice. ^#^*P*<0.05, ^##^ *P*<0.05 vs. control with same intervention.
