# Supplementary material for: Sustained Toll-Like Receptor 9 Activation Promotes Systemic and Cardiac Inflammation, and Aggravates Diastolic Heart Failure in SERCA2a KO Mice
Source: PLoS One. 2015 Oct 13;10(10):e0139715. doi: 10.1371/journal.pone.0139715 (PMC4604200; doi:10.1371/journal.pone.0139715)
Supplement: S1 Methods — (DOC) [file pone.0139715.s004.doc]

Supporting Methods

### Phase contrast magnetic resonance imaging (PC-MRI)

Anesthesia was maintained by administration of a mixture of oxygen and approximately 1.5 % isoflurane in spontaneously breathing animals. Heart rate, body temperature and respiration rate were constantly monitored during experiments, and the level of anesthesia was slightly regulated to keep the heart and respiration rate as stable as practically possible. Two sub dermal ECG-electrodes were positioned to monitor heart rate, and a respiration pillow placed on the abdomen was used to monitor respiration. In addition, body temperature was maintained by heated air and monitored with a rectal temperature probe placed for continuous surveillance. As contrast solution, we injected animals with Gadolinium contrast agent, Magnevist (Bayer), i.p. PC-MRI acquisition was performed using an RF-spoiled black-blood gradient echo cine sequence employing nine-point balanced encoding. Two planes of mid-ventricular and four-chamber were imaged, covering >100% of the R-R-interval. PC-MRI imaging parameters were: TR (repetition time)=3.1 ms, TE (echo-time) =2.3 ms, field-of-view=25,6 mm x 25,6 mm, matrix 96 x 96, slice thickness = 1.0 mm, average = 1; flip angle= 12 °C, venc = 8 cm/s PC-MRI experiments were ECG-triggered and respiration gated. The total experiment duration was 75 min per animal. MRI data was analyzed using Matlab (The MathWorks, Natick, MA) with the operator blinded to animal groups. All cardiac imaging was recorded by an investigator blinded to the treatment groups.
