# Supplementary material for: Sustained Toll-Like Receptor 9 Activation Promotes Systemic and Cardiac Inflammation, and Aggravates Diastolic Heart Failure in SERCA2a KO Mice
Source: PLoS One. 2015 Oct 13;10(10):e0139715. doi: 10.1371/journal.pone.0139715 (PMC4604200; doi:10.1371/journal.pone.0139715)
Supplement: S1 Table — (DOC) [file pone.0139715.s005.doc]

Supporting Tables

| Fur appearance | Normal=0 | Reduced grooming=1 | Untidy fur=2 | Very untidy fur=4 |
| --- | --- | --- | --- | --- |
| Behavior | Normal=0 | Reduced activity=1 | Very reduced activity=2 |  |
| Body weight loss | Normal=0 | Loss<5%=1 | Loss<10%=2 | Loss<15%=3 |
| Movement | Normal=1 | Unsteady walk=2 | Circulating walk=3 | Cannot move=4 |
| Euthanization |  |  |  | If total score is> 8 |

S1 Table. Pre-specified criteria for evaluating morbidity and spontaneous death
