# Supplementary material for: Sustained Toll-Like Receptor 9 Activation Promotes Systemic and Cardiac Inflammation, and Aggravates Diastolic Heart Failure in SERCA2a KO Mice
Source: PLoS One. 2015 Oct 13;10(10):e0139715. doi: 10.1371/journal.pone.0139715 (PMC4604200; doi:10.1371/journal.pone.0139715)
Supplement: S2 Table — (DOC) [file pone.0139715.s006.doc]

| **Heart** | | |  |  |  |
| --- | --- | --- | --- | --- | --- |
| **Cardiac cell stress and/or death** | | |  |  |  |
| Nuclear-to- cytoplasm ratio | Score | Vacuolization or necrotic muscle fibres | Score | Interstitial inflammation | Score |
| Absent-small nuclear variation | 0 | Absent | 0 | Absent | 0 |
| Moderate nuclear variation | 1 | Rare or occasional | 1 | Rare or occasional | 1 |
| Marked nuclear variation with nucleoli detectable with 100x magnification | 2 | Several per field of vision in 200x magnification | 2 | Several per field of vision in 200x magnification | 2 |
| Light muscle fibres with swollen nucleus and increased nucleoli | 3 |  |  |  |  |
| Many cells as score 3 | 4 |  |  |  |  |
| **Lung** |  |  |  |  |  |
| Vascular inflammation | Score | Alveolar inflammation | Score |  |  |
| Absent | 0 | Absent | 0 |  |  |
| Less than 3 cell layers | 1 | Focal | 1 |  |  |
| More than 3 cell layers | 2 | Marked | 2 |  |  |
| Additional inflammation in intima | 3 |  |  |  |  |
| **Liver** |  |  |  |  |  |
| Portal inflammation | Score | Lobular inflammation | Score |  |  |
| Absent | 0 | Absent | 0 |  |  |
| Focal and minimal | 1 | Rare and small foci | 1 |  |  |
| Mild | 2 | Occasional small foci | 2 |  |  |
| Moderate | 3 | Moderate small foci | 3 |  |  |
| Marked | 4 | Frequent small foci | 4 |  |  |

Supporting Tables

S2 Table. Eight weeks after gene excision and 4 weeks after initiation of sustained TLR9 stimulation, the degree of inflammation in hearts, lungs and livers were scored by a pathologist blinded to genotype and intervention
