# Supplementary material for: Sustained Toll-Like Receptor 9 Activation Promotes Systemic and Cardiac Inflammation, and Aggravates Diastolic Heart Failure in SERCA2a KO Mice
Source: PLoS One. 2015 Oct 13;10(10):e0139715. doi: 10.1371/journal.pone.0139715 (PMC4604200; doi:10.1371/journal.pone.0139715)
Supplement: S3 Table — (DOC) [file pone.0139715.s007.doc]

Supporting Tables

S3 Table. Primer sequences used in RT PCR analyses.

| **Target** | **Sequence (5’🡪3’**) | **Acc.nr** |
| --- | --- | --- |
| CXCL10 | (+)-GCCATAGGGAAGCTTGAAATCA | NM_021274 |
|  | (-)-ATCATTCTTTTTCATCGTGGCAAT |  |
| CXCL2 | (+)-CCCAGACAGAAGTCATAGCCACT | NM_009140 |
|  | (-)-TGCCTTTGTTCAGTATCTTTTGGA |  |
| MCP-1 | (+)-AAAGAAGCTGTAGTTTTTGTCACCAA | NM_011333 |
|  | (-)-TTAATGTATGTCTGGACCCATTCCT |  |
| TNF-α | (+)-AGACCCTCACACTCAGATCATCTTC | NM_013693 |
|  | (-)-CCACTTGGTGGTTTGCTACGA |  |
| Collagen 1 | (+)-CCTGAGTCAGCAGATTGAGAACA  (-)-TCGATCCAGTACTCTCCGCTCT | NM_007742 |
| Collagen 3 | (+)-TCTATGAATGGTGGTTTTCAGTTCA | NM_009930 |
|  | (-)-TTTTTGCAGTGGTATGTAATGTTCTG |  |
| GAPDH | (+)-CCAAGGTCATCCATGACAACTT | NM_008084 |
|  | (-)-AGGGGCCATCCACAGTCTT |  |

CXCL10, Chemokine C-X-C motif ligand 10; CXCL2, Chemokine C-X-C motif ligand 2; MCP-1, Monocyte chemotactic protein-1; TNF-α, tumor necrosis factor-α; GAPDH, glyceraldehydes 3-phosphate dehydrogenase; Acc.nr.,GenBank Accession number; (+): forward primer, (-): reverse primer.
