# Supplementary material for: Sustained Toll-Like Receptor 9 Activation Promotes Systemic and Cardiac Inflammation, and Aggravates Diastolic Heart Failure in SERCA2a KO Mice
Source: PLoS One. 2015 Oct 13;10(10):e0139715. doi: 10.1371/journal.pone.0139715 (PMC4604200; doi:10.1371/journal.pone.0139715)
Supplement: S4 Table — (DOC) [file pone.0139715.s008.doc]

Supporting Tables

**S4 Table. Physiological parameters in SERCA2a KO and control mice 8 weeks after gene excision and 4 weeks after initiation of TLR9 stimulation.**

|  | **Controls** | | **SERCA2a KO** | |
| --- | --- | --- | --- | --- |
|  | **PBS (n=7)** | **CpG B (n=4-7)** | **PBS (n=8-12)** | **CpG B (n=5-8)** |
| TL | 19.1 ± 0.7 | 18.5 ± 1.0 | 19.4 ± 0.9 | 19.5 ± 0.9 |
| BW(g) | 29.2 ± 2.0 | 30.2 ± 2.1 | 31.0 ± 1.4 | 31.3 ± 1.8 |
| HW(mg)/TL | 7.5 ± 1.4 | 8.1 ±1.2 | 8.4± 0.5 | 8.7± 0.9 |

BW, Body weight; HW, Heart weight; TL, Tibia length (mm). Data are expressed as the mean±SD. Statistics were done using Two-way ANOVA test, followed by Mann Whitney U- test.
